# *In vitro* evaluation of antibacterial activity of phytochemicals and micronutrients against *Borrelia burgdorferi* and *Borrelia garinii*

**DOI:** 10.1111/jam.12970

**Published:** 2015-11-22

**Authors:** A. Goc, A. Niedzwiecki, M. Rath

**Affiliations:** ^1^ Dr. Rath Research Institute BVSanta ClaraCAUSA

**Keywords:** biofilm, *Borrelia* sp., cysts, Lyme disease, phytochemicals, spirochetes

## Abstract

**Aims:**

Little is known about the effects of phytochemicals against *Borrelia* sp. causing Lyme disease. Current therapeutic approach to this disease is limited to antibiotics. This study examined the anti‐borreliae efficacy of several plant‐derived compounds and micronutrients.

**Methods and Results:**

We tested the efficacy of 15 phytochemicals and micronutrients against three morphological forms of *Borrelia burgdoferi* and *Borrelia garinii*: spirochetes, latent rounded forms and biofilm. The results showed that the most potent substances against the spirochete and rounded forms of *B. burgdorferi* and *B. garinii* were cis‐2‐decenoic acid, baicalein, monolaurin and kelp (iodine); whereas, only baicalein and monolaurin revealed significant activity against the biofilm. Moreover, cis‐2‐decenoic acid, baicalein and monolaurin did not cause statistically significant cytotoxicity to human HepG2 cells up to 125 *μ*g ml^−1^ and kelp up to 20 *μ*g ml^−1^.

**Conclusions:**

The most effective antimicrobial compounds against all morphological forms of the two tested *Borrelia* sp. were baicalein and monolaurin. This might indicate that the presence of fatty acid and phenyl groups is important for comprehensive antibacterial activity.

**Significance and Impact of the Study:**

This study reveals the potential of phytochemicals as an important tool in the fight against the species of *Borrelia* causing Lyme disease.

## Introduction

Lyme disease (LM, *Lyme borreliosis*) is a zoonotic infectious disease that has become a worldwide threat for humans and animals (Burgdorfer *et al*. 1982; Dryden and Hodgkins 2010; Johnson *et al*. 2014). This disease is transmitted by ticks of the genus *Ixodes* (Benach *et al*. 1983; Steere *et al*. 1983), which harbor the *Borrelia* sp. bacteria and spread them when feeding on reservoir animals such as reptiles, birds, and small and large mammals (Benach *et al*. 1983; Salkeld and Lane 2010; Richter *et al*. 2011; Stricker and Johnson 2011; Norte *et al*. 2013). These pathogens are now documented in the northeastern, mid‐Atlantic, north‐central and western Pacific coasts of the United States, as well as in Europe, Asia, Africa, and Australia (Burgdorfer and Keirans 1983; Bacon *et al*. 2008; Hubalek 2009). People of all ages and both genders are equally at risk, although the highest rates of infection have been reported in children ages 10–14 and in adults ages 45 and older (Strle *et al*. 2013; Robinson 2014). The newest estimates, which are calculated based on diagnostic test results and insurance records, indicate that the number of Lyme disease cases in the United States alone reaches 300 000 each year. Based on physician reporting, it is estimated to be ca. 30 000 new cases per year. There are unreported cases, however, that are not reflected in the statistics (Johnson *et al*. 2014; Stricker and Johnson 2014).

The causative agent of LM was established by Willy Burgdorfer who identified *Borrelia burgdorferi* as a pathogenic factor (Burgdorfer 1991; Stricker and Johnson 2014). Today we know that two more species, *Borrelia afzelii* and *Borerlia garinii,* are involved as well (Lovrich *et al*. 1994; Rudenko *et al*. 2011). *Borrelia* are micro‐aerophilic and slow‐growing spirochetal bacteria classified in 36 known species with unknown numbers of genomic strains. Although the clinical symptoms of infection with different species may vary, common indicators have been identified (Vanousova and Hercogova 2008; Murray and Shapiro 2010). Early signs of LM occur within a month after tick's bite and are indicated by skin lesion (described by some as a redness or rash with a bull's eye pattern). This lesion called erythema migrans is one of the hallmarks of Lyme disease. It is often accompanied by fever, fatigue, body aches and headache (Steere 1989; Burgdorfer 1991; Shapiro 2014). Approximately 4–6 weeks or months after the first symptoms appear, the systemic signs may surface and are much more severe with musculoskeletal indications, neurologic problems, cardiac abnormalities, and eye and liver inflammation (Burgdorfer 1991; Stanek *et al*. 2012; Shapiro 2014). Interestingly, not all Lyme patients have all the symptoms. Asymptomatic infection has been observed, but it occurs in <10% of patients in the United States (Steere *et al*. 2003; Stricker and Phillips 2003).

A few antimicrobial compounds (i.e. mainly synthetic antibiotics) have been scientifically examined against *Borrelia* sp. (Brorson and Brorson 2006; Brorson *et al*. 2009; Sapi *et al*. 2011; Kadam *et al*. 2014). To date, there are several FDA‐approved antibiotic‐related treatments used as a primary approach (guideline) for patients (Klempner *et al*. 2013; Cameron *et al*. 2014; Shapiro 2014). For early stages of Lyme disease, administration of doxycycline is generally the first choice; however, this antibiotic cannot be taken by children and pregnant or breastfeeding women (Loewen *et al*. 1999; Hansmann 2009). Other alternatives include amoxicillin, and cefuroxime (Loewen *et al*. 1999; Stricker *et al*. 2004; Larkin 2008; Hansmann 2009; Shapiro 2014), and for late stages of the disease ceftriaxone or cefotaxime (Maraspin *et al*. 2011; Delong *et al*. 2012) are administrated. Unfortunately, continued antibiotic treatment is not recommended as its long‐term effectiveness has not been observed or proven (Delong *et al*. 2012; Stanek *et al*. 2012). The increasing trend with new and relapsing Lyme disease cases was noted and attributed mainly to inadequate prevention, ineffective therapy and/or bacterial persistency (Straubinger *et al*. 1997; Bockenstedt *et al*. 2002; Peltomaa *et al*. 2003; Hodzic *et al*. 2008; Feng *et al*. 2014).

The efficacy of natural plant‐derived chemicals and micronutrients, including a variety of essential oils, vitamins, and plant metabolites, as anti‐borreliaea agents is still not well known. (Zhang *et al*. 2013; Morrison and Hergenrother 2014). To date, only a few plant metabolites have undergone extensive scientific evaluation for antimicrobial activity against *Borrelia* sp., e.g. grape seed extract and teasel root extract (Brorson and Brorson 2007; Liebold *et al*. 2011). There is an enormous potential in exploring anti‐borreliae properties of natural substances as they are generally ascribed as safe with promising outcomes, and they might be effective as a substitution or adjunct treatment to the standard antibiotic based therapies (Patil and Saraogi 2014; Takeuchi *et al*. 2014).

In this study, we tested the efficacy of 15 phytochemicals and micronutrients against different forms of *B. burgdorferi* and *B. garinii* (i.e. spirochetes, rounded forms and biofilm). These compounds were selected from naturally occurring (nonsynthetic) and plant‐derived substances with potential anti‐bacterial properties that have not yet been scientifically evaluated against *Borrelia* sp. Also, the selection was limited to compounds with validated safety in *in vivo* studies and with chemical structure suggesting effectiveness against all morphological forms of *Borrrelia* sp. To our knowledge this is the first attempt to provide such a comprehensive evaluation of naturally occurring compounds against *Borrelia* sp., which could advance our knowledge about their antibacterial properties, and help in the development of new approaches or improve already existing treatments for Lyme disease.

## Materials and methods

### Test compounds

The following compounds, with purity between 90–98% according to the manufacturer, were obtained from Sigma (St. Louis, MO): hydroxytyrosol, baicalein, cis‐2‐decenoic acid, morin, oenin, vitamin D3 (1,25‐dihydroxycholecalciferol) and vitamin C (ascorbic acid). The following compounds, with purity between 97–99% according to the manufacturer, were purchased from Tocris Bioscience (Bristol, UK): rosmarinic acid, kaempferol, piceatannol, rottlerin, luteolin and fisetin. Other reagents used in this study were organic kelp with standardized iodine content (i.e. 150 *μ*g ml^−1^ as 100% Daily Value) purchased from World Organic Ltd. (Auckland, New Zealand), and monolaurin (Lauricidin^®^) purchased from Med‐Chem Laboratories, Inc., (Goodyear, AZ) as a pure sn‐1 monolaurin (glycerol monolaurate) derived from coconut oil.

### Preparation of test compounds for susceptibility testing

A stock solution (50–100 mg ml^−1^) of all compounds (depending on solubility of each substance) was prepared by suspending each test compound in absolute ethanol and sterilizing it by 0·22 *μ*m syringe filtration. All stock solutions were stored in aluminium foil‐wrapped tubes at −20°C. As ethanol could be bactericidal, the amount of ethanol added to growth medium was kept as low as possible. A preliminary experiment determined that its content should not exceed 0·5% (v/v) (data not shown). Therefore, in the experiments the final concentration of ethanol in growth medium was kept below 0·4% (v/v). The appropriate amounts of each stock solution were added to 1·8 ml sterile screw‐cap test tubes containing 1 ml of BSH complete medium to yield final concentrations of 50–1000 *μ*g ml^−1^ for all compounds. Ethanol at 0·1–0·4% (v/v) was applied as negative control. Doxycycline at 5–500 *μ*g ml^−1^ concentration range was used as positive control.

### Test micro‐organisms

Two *Borrelia* species, *B. burgdorferi* and *B. garinii*, were tested in their three morphological forms: spirochetes, rounded forms, and biofilm. As *Borrelia* sp. are aero‐tolerant anaerobes, they were cultured stationary in the presence of 5% CO_2_ in screw‐capped tubes. Low passage isolates of the B31 strain of *B. burgdorferi* and CIP103362 strain of *B. garinii* were obtained from the American Type Culture Collection (Manassas, VA). Stocks of both species were cultured in commonly applied conditions, i.e. Barbour‐Stoner‐Kelly H (BSK‐H) medium, supplemented with 6% rabbit serum (Sigma, St. Louis, MO) without antibiotics at 33°C with 5% CO_2_, in sterile screw‐capped 15 ml polypropylene test tubes with or without gentle shaking. B31 strain is an isolate from *Ixodes dammini*, whereas CIP103362 strain is an isolate from *Ixodes ricinus*.

### Preparation of test micro‐organisms for susceptibility testing

*Borrelia burgdorferi* and *B. garinii* strains were prepared for testing as described by Sapi, *et al*. (Sapi *et al*. 2011). Briefly, the strains were activated from original cryobank vials and inoculated into 10 ml BKS‐H complete medium, and maintained at 33°C. For generation of homogeneous cultures (i.e. having only spirochetal form) of *B. burgdorferi* or *B. garinii,* spirochetes were inoculated and maintained at 33°C in a shaking incubator at 250 rev min^−1^, so there was no biofilm formation (Sapi *et al*. 2011). To generate biofilm‐like colonies of *B. burgdorferi* or *B. garinii*, the spirochetes were inoculated in four‐well chambers (BD Biosciences, Sparks, MD) coated with rat‐tail collagen type I and incubated for 1 week without shaking.

### Evaluation of bacteriostatic effect of test compounds on test micro‐organisms

Growth of *B. burgdorferi* and *B. garinii* was tested using a macro‐dilution method according to Sapi, *et al*. (Sapi *et al*. 2011). Briefly, 1·8 ml sterile screw‐capped test tubes containing 1 ml BSK‐H medium, supplemented with the test compound of interest were inoculated with 2 × 10^6^ spirochetes ml^−1^ of the homogenous bacterial suspension. The tubes were then incubated at 33°C and growth was monitored at regular intervals for up to 72 h. The entire experiment was repeated three times for each bacteria strain and each concentration of the tested compounds. Control cultures were treated with ethanol (0·1–0·4 v/v) alone or doxycycline (5–500 *μ*g ml^−1^). Bacterial growth was assessed by a bacterial Petroff‐Hausser counting chamber for up to 72 h of incubation using dark field microscopy (direct cell counting) as a standard procedure.

### Evaluation of bactericidal effect of test compounds on test micro‐organisms

Bactericidal effect of tested compounds was examined using a fluorescence method according to Sapi, *et al*. (Sapi *et al*. 2011). Briefly, 2 × 10^6^ spirochetes ml^−1^ of the homogenous bacterial suspension was inoculated into each 1·8 ml sterile screw‐capped test tube containing 1 ml BSK‐H medium, supplemented with the test compound of interest. Control cultures were treated with ethanol (0·1–0·4 v/v) alone or doxycycline (5–500 *μ*g ml^−1^). The tubes were then incubated at 33°C and viability was monitored at regular intervals for up to 72 h. The entire experiment was repeated three times for each strain and each concentration. The susceptibility of rounded forms to the test compound was then assessed up to 72 h by LIVE/DEAD^®^ BacLight^™^ Bacterial Viability Assay using a fluorescence microscope (Nikon, Eclipse E600, Melville, NY) and the percentage of live (green) and dead (red) *B. burgdorferi* and *B. garinii* rounded forms was calculated.

### Evaluation of test compounds on bacterial biofilm

The qualitative effect of the test compounds against biofilm‐like colonies of *B. burgdorferi* and *B. garinii* was evaluated using the commonly used crystal violet (CV) staining method, according to Sapi, *et al*. (Sapi *et al*. 2011). Briefly, 1 × 10^7^ spirochetes ml^−1^ of homogeneous bacterial suspension were inoculated into collagen type I‐coated four‐well chambers and incubated for 1 week. Subsequently, biofilm‐like colonies were treated with various concentrations of the test compounds. Control wells were treated with ethanol (0·1–0·4 v/v) alone or doxycycline (5–500 *μ*g ml^−1^). All chambers were then incubated at 33°C for up to 72 h. Next, for quantitative analysis, all wells were fixed with 500 *μ*l of cold methanol‐formalin (1 : 1) for 30 min. and stained with 1 ml of CV (0·1%) for 10 min. The biofilms were washed carefully three times with 1× PBS (phosphate‐buffered saline), and 1 ml of methanol was added to each well to extract a dye which was measured at 595 nm (Molecular Device, Spectra Max 340, Sunnyvale, CA). Each experiment was repeated three times for each strain and each compound concentration. For qualitative analysis, all wells were fixed with 500 *μ*l of cold formalin‐acetic acid mixture for 20 min. followed by staining with 200 *μ*l of 2× BacLight staining mixture for 15 min. in the dark, according to the manufacturer's recommendation. Pictures were immediately taken from untreated (control) and treated mounted slides using a fluorescence microscope (Nikon, Eclipse E600). Immunofluorescence staining with primary antibodies against *B. burgdorferi* (Abcam, Cambridge, MA) and *B. garinii* (Santa Cruz, Santa Cruz, CA) with AlexaFluor‐488 as a secondary antibody was performed accordingly to manufactures' recommendation respectively.

### Evaluation of cytotoxicity of tested compounds on human cells

Cells viability was assessed using MTT assay according to the manufacturer's protocol. Briefly, human HepG2 cell line was plated in 48‐well plates at 1 × 10^5^ cells per well in the DMEM containing 10% FBS. After 24 h, the medium was replaced with the same medium supplemented with the following compounds: cis‐2‐decenoic acid (125–1000 *μ*g ml^−1^), monolaurin (125–1000 *μ*g ml^−1^), baicalein (125–1000 *μ*g ml^−1^), and kelp (iodine) (5–50 *μ*g ml^−1^). After 72 h of treatment, cells viability was measured at 570 nm, using an ELISA reader (Molecular Device, Spectra Max 340). The experiment was repeated four times for each strain and each compound concentration.

### Statistical analysis

All data are presented as means ± SD (*n* = 3). The Student's two‐tailed *t* test was used to determine statistically significant differences set at 0·05 levels. Statistical analysis was performed using graphpad software (La Jolla, CA).

## Results

### Effect of tested compounds on spirochetes of *Borrelia* sp

**Figure 1:**
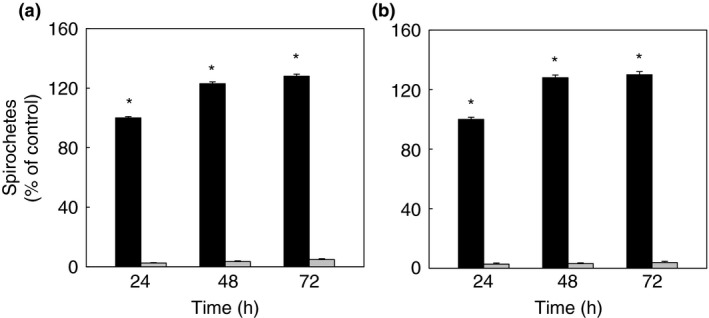
Kinetic evaluation of growth of live/dead untreated spirochetes of *Borrelia burgdorferi* B31 strain (a) and *Borrelia garinii* CIP103362 strain (b) monitored up to 72 h. Live spirochetes (■), dead spirochetes (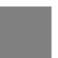), *&!blank;*P* ≤ 0·001.

The effect of 15 selected natural compounds on the growth of spirochete forms of *B. burgdorferi* (B31 strain) and *B. garinii* (CIP103362 strain) was examined and presented as the minimal inhibitory (MIC) and the minimal bactericidal (MBC) concentrations in Table 1. The results show that all compounds expressed anti‐spirochetal activity with MIC values ranging from 50 to 250 *μ*g ml^−1^ for phytochemicals, and from 0·0005 to 35 *μ*g ml^−1^ for the micronutrients. The MBC values oscillated between 200 and 500 *μ*g ml^−1^ for phytochemicals, and from 0·002 to 88 *μ*g ml^−1^ for the micronutrients. The MIC values for fisetin, kaemferol, rosmarinic acid, baicalein, monolaurin, morin, piceatannol, rottlerin, vitamin D3 and kelp (iodine) revealed their anti‐spirochetal potential at the same concentrations in both tested species of *Borreli*; whereas, other substances such as hydroxytyrosol, oenin, cis‐2‐decenoic acid, luteolin and vitamin C were effective at lower concentrations against *B. burgdorferi* and higher concentrations against *B. garinii*. The MBC values of all tested compounds corresponded to each other for both tested *Borrelia* sp. Antibiotic doxycycline was used as positive control with the MIC value established as 25 *μ*g ml^−1^ and the MBC value as 200 *μ*g ml^−1^ for *B. burgdorferi* and 250 *μ*g ml^−1^ for *B. garinii*. These values were in agreement with those reported by Sapi, *et al*. (Sapi *et al*. 2011). Kinetic growth and viability of untreated spirochetes of both tested species of *Borrelia* was not affected up to 72 h (Fig. 1).

**Table 1:** Antibacterial activity of 15 tested compounds against spirochete form of *Borrelia burgdorferi* B31 and *Borrelia garinii* CIP103362

Tested compound	*B. burgdorferi*		*B. garinii*	
	MIC (*μ*g ml^−1^)	MBC (*μ*g ml^−1^)	MIC (*μ*g ml^−1^)	MBC (*μ*g ml^−1^)
Hydroxytyrosol	50	250	100	250
Fisetin	125	250	125	250
Kaemferol	200	300	200	300
Oenin	75	200	100	200
Cis‐2‐decenoic acid	125	250	250	250
Rosmarinic acid	150	250	150	250
Luteolin	125	250	150	250
Baicalein	150	250	150	250
Monolaurin	100	250	100	250
Morin	100	250	100	250
Piceatannol	250	500	250	500
Rottlerin	125	250	125	250
Vitamin D3	0·0005	0·001	0·0005	0·001
Vitamin C	35	88	88	88
Kelp (Iodine)	5	15	5	15
Doxycycline	25	200	25	250

MIC, minimal inhibitory concentration; MBC, minimal bactericidal concentration.

### Effect of tested compounds on rounded forms of *Borrelia* sp

The bactericidal effects of 15 selected natural compounds on latent rounded forms of *B. burgdorferi* (B31 strain) and *B. garinii* (CIP103362 strain) were examined and presented as LD_50_ values in Table 2. The results show that none of the tested compounds could eliminate rounded forms in 90–99%, up to their maximum tested concentration (i.e. 1000 *μ*g ml^−1^). The MBC determination method is a very rigorous requirement even for many antibiotics. However, compounds such as hydroxytyrosol, cis‐2‐decenoic acid, baicalein and monolaurin were able to induce dead of latent rounded forms at LD_50_ values between 300 and 500 *μ*g ml^−1^, and at 20 *μ*g ml^−1^ for kelp (iodine). Moreover, their bactericidal potential towards rounded forms was observed at the same concentrations for both tested species of *Borrelia*. Doxycycline was used as positive control in this experiment. This antibiotic could not eliminate rounded forms in 90–99% or achieve LD_50_ level, even at the maximum tested concentration of 500 *μ*g ml^−1^. These values stay in agreement with those reported by Sapi, *et al*. (Sapi *et al*. 2011).

**Table 2:** Antibacterial activity of 15 tested compounds against latent rounded forms of *Borrelia burgdorferi* B31 and *Borrelia garinii* CIP103362

Tested compound	*B. burgdorferi*	*B. garinii*
	LD_50_ (*μ*g ml^−1^)	LD_50_ (*μ*g ml^−1^)
Hydroxytyrosol	300	300
Fisetin	NS	NS
Kaemferol	NS	NS
Oenin	NS	NS
Cis‐2‐decenoic acid	500	500
Rosmarinic acid	NS	NS
Luteolin	200	NS
Baicalein	350	350
Monolaurin	300	300
Morin	NS	NS
Piceatannol	NS	NS
Rottlerin	NS	NS
Vitamin D3	NS	NS
Vitamin C	NS	NS
Kelp (Iodine)	20	20
Doxycycline	NS	NS

LD_50_, lethal dose causing 50% of killing; NS, not susceptible at the maximal tested concentration.

### Antibacterial potential of tested compounds on *Borrelia* sp. biofilm

**Figure 2:**
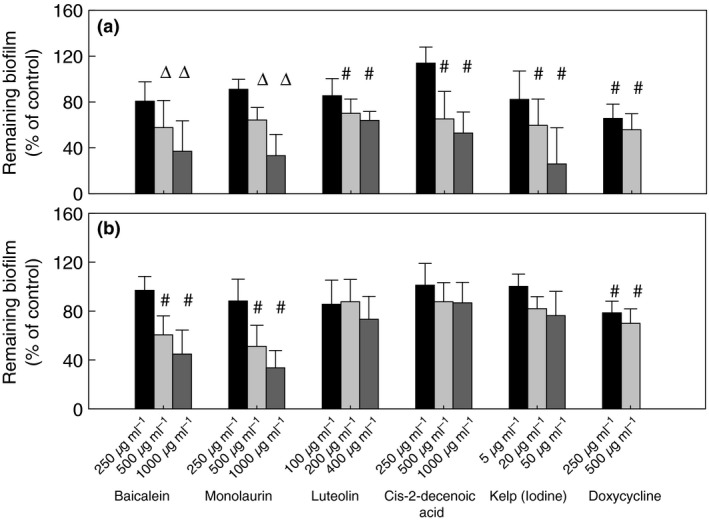
Dose‐dependent estimation of remaining biofilm of *Borrelia burgdorferi* B31 strain (a) and *Borrelia garinii* CIP103362 strain (b) after 72 h of post‐treatment with phytochemicals and antibiotic doxycycline presented as a quantitative examination; #*P* ≤ 0·05, ∆*P* ≤ 0·01.

**Figure 3:**
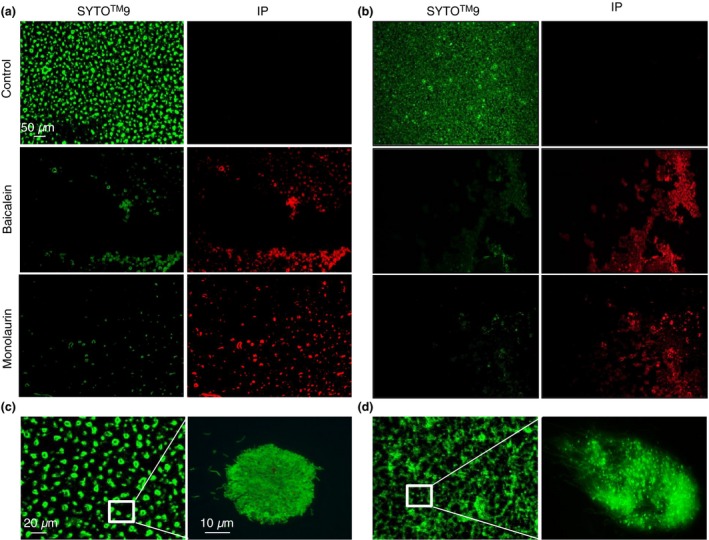
Evaluation of untreated (control) and treated with Baicalein (500 *μ*g ml^−1^) or Monolaurin (500 *μ*g ml^−1^) biofilm of *Borrelia burgdorferi* B31 strain (a) and *Borrelia garinii* CIP103362 strain (b) after 72 h as a qualitative measurement using LIVE/DEAD BacLight staining; green – live biofilm, red – dead biofilm, images taken at 4 × magnification. Immunofluorescence staining with primary antibodies against untreated *B. burgdorferi* B31 strain (c) and *B. garinii* CIP103362 strain (d) after 72 h using AlexaFluor‐488 as a secondary antibody respectively; images taken at 10× (right) and 63× (left) magnification.

The effect of 15 selected natural compounds on biofilm formed by *B. burgdorferi* (B31 strain) and *B. garinii* (CIP103362 strain) are presented on Fig. 2. The results show that five compounds such as baicalein (≥500 *μ*g ml^−1^), luteolin (≥200 *μ*g ml^−1^), monolaurin (≥500 *μ*g ml^−1^), cis‐2‐decenoic acid (≥500 *μ*g ml^−1^) and kelp (iodine) (≥20 *μ*g ml^−1^), could reduce biofilm‐like colonies formed by *B. burgdorferi* (B31 strain). Only two compounds, baicalein (≥500 *μ*g ml^−1^) and monolaurin, (≥500 *μ*g ml^−1^), were effective against biofilm formed by *B. garinii* (CIP103362 strain). Baicalein and monolaurin reduced *B. burgdorferi* biofilm‐like colonies by approx. 30–60% and *B. garinii* by approx. 40–60%. In addition, kelp (iodine), cis‐2‐decenoic acid and luteolin significantly reduced biofilm‐like colonies formed by *B. burgdorferi,* but displayed only a decreasing tendency towards biofilm formed by *B. garinii*. We observed that biofilm‐like colonies formed by *B. burgdorferi* appeared as loose in consistency and smaller compared to those seen in control. However, biofilm formed by *B. garinii* treated with monolaurin or baicalein looked similar to the control except it was more detachable (Fig. 3). The antibiotic doxycycline (positive control) reduced biofilms formed by both tested *Borrelia* sp. by about 40%. These results were in agreement with those reported by Sapi, *et al*. (Sapi *et al*. 2011).

### Bactericidal kinetic of tested compounds

**Figure 4:**
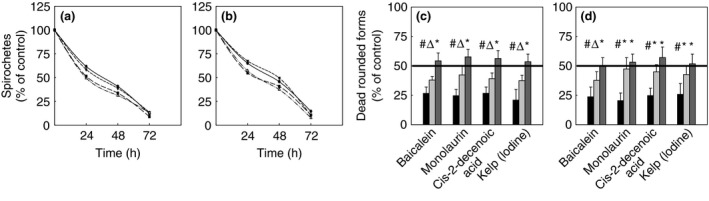
Kinetic evaluation of bactericidal effect of phytochemicals against the spirochetes (a and b) and rounded forms (c and d) of *Borrelia burgdorferi* B31 strain (a and c) and *Borrelia garinii* CIP103362 strain (b and d) monitored up to 72 h. (a and b) All data are statistically significant; tested compounds: 250 *μ*g ml^−1^ Baicalein (▼), 250 *μ*g ml^−1^ Monolaurin (∆), 250 *μ*g ml^−1^ Cis‐2‐decenoic acid (●), 20 *μ*g ml^−1^ Kelp (Iodine) (□). (c and d) LD50 marked with the solid line; #*P* ≤ 0·05, ∆*P* ≤ 0·01, *P ≤ 0·001; tested compounds: 350 *μ*g ml^−1^ Baicalein, 300 *μ*g ml^−1^ Monolaurin, 500 *μ*g ml^−1^ Cis‐2‐decenoic acid, 20 *μ*g ml^−1^ Kelp (Iodine); 24 h (■), 48 h (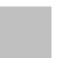), 72 h (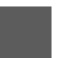).

We evaluated the kinetic of the bactericidal effect of cis‐2‐decenoic acid, baicalein, monolaurin and kelp (iodine) as these compounds were most effective in eliminating spirochetes and latent rounded forms of both tested *Borrelia* sp. The results presented in Fig. 4a,b show that these phytochemicals acted in a time‐dependent manner in decreasing spirochete forms of *B. burgdorferi* and *B. garinii*. A time‐dependent killing effect on their latent rounded forms was also observed that oscillated between 20–25% after 24 h, 30–40% after 48 h and 50–55% after 72 h of exposure, compared to controls (Fig. 4c,d).

### Cytotoxicity

**Figure 5:**
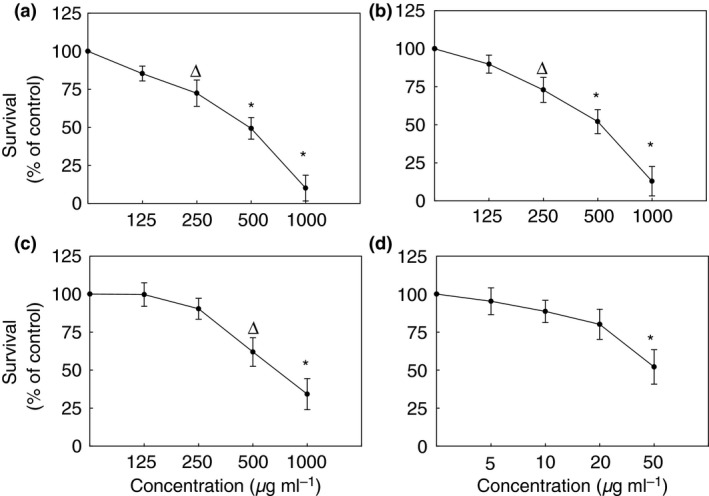
Dose‐dependent cytotoxic effect of phytochemicals on HepG2 cells assessed by MTT assay after 72 h; #*P* ≤ 0·05, ∆*P* ≤ 0·01, *P ≤ 0·001. Tested compounds: 125–1000 *μ*g ml^−1^ Baicalein (a), 125–1000 *μ*g ml^−1^ Monolaurin (b), 125–1000 *μ*g ml^−1^ Cis‐2‐decenoic acid (c), 5–50 *μ*g ml^−1^ Kelp (Iodine) (d).

**Figure 6:**
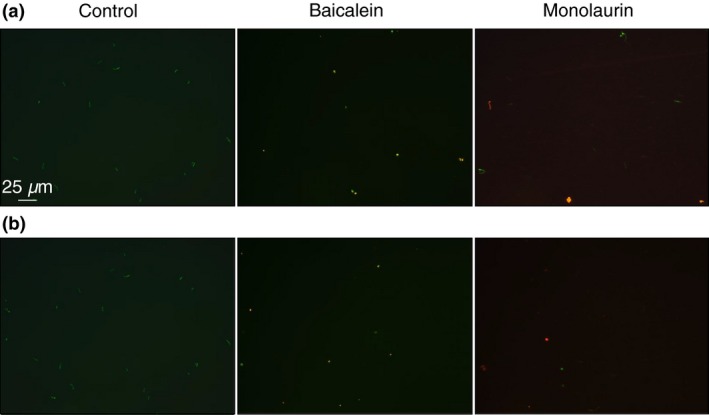
Evaluation of untreated (control) and treated with Baicalein (350 *μ*g ml^−1^) or Monolaurin (300 *μ*g ml^−1^) *Borrelia burgdorferi* B31 strain (a) and *Borrelia garinii* CIP103362 strain (b) after 72 h as a qualitative measurement by fluorescence microscope using LIVE/DEAD BacLight staining; green fluorescence – live organisms (spirochetes and rounded forms), red fluorescence – dead organisms (spirochetes and rounded forms). Merged images taken at 20× magnification.

Cytotoxic evaluation results presented in Fig. 5 show that cis‐2‐decenoic acid, baicalein and monolaurin did not display significant cytotoxicity towards human HepG2 cells up to 125 *μ*g ml^−1^ and caused about 30% decrease in cells survival at 250 *μ*g ml^−1^. Their 50% of cytotoxic concentration (CC_50_) was found to be near 500 *μ*g ml^−1^. Kelp (iodine) was nontoxic at 20 *μ*g ml^−1^ and 50% of cytotoxic concentration (CC_50_) at 50 *μ*g ml^−1^ (Fig. 5). For comparison, baicalein and monolaurin have shown to be effective against all morphological forms of both tested *Borrelia* sp. (Fig. 6).

## Discussion

Our study demonstrates the *in vitro* susceptibility of different morphological forms of *B. burgdorferi* (prevalent in North America), as well as *B. garinii* (prevalent in Europe), to various nonsynthetic plant‐derived compounds and micronutrients. It has been suggested that to successfully eliminate *Borrelia* sp., the antimicrobial agents ought to be effective against their vegetative and viable atypical (latent) forms (i.e. rounded forms and biofilm) (Sapi *et al*. 2011). The vegetative form of *Borrelia* sp. are spirochetes that are active and motile and can survive viscous conditions in human and animal bloodstreams and penetrate tissues and cells (Groshong and Blevins 2014; Miller *et al*. 2014). Any hostile conditions, such as changes in temperature, pH, starvation, antibiotic exposure or threats from the host's immune system, can alter their structure converting them to the latent rounded forms (i.e. cysts, granular forms and spheroplats/CDW forms) and/or biofilm. Presence of these atypical forms might attribute to persistency of *Borrelia* sp. in the human body even for decades (Brorson and Brorson 1997; Alban *et al*. 2000; Gruntar *et al*. 2001; Stewart and Costerton 2001; Jefferson 2004; Murgia and Cinco 2004), although further studies are warranted. The detection of only rounded forms of *B. burgdorferi* in human specimens indicates the need for further studies assessing a relationship between the presence of atypical forms of *Borrelia* sp. and Lyme disease, in its both typical and persistent clinical forms (Lantos *et al*. 2014).

Our study has shown that the most promising compounds effective against spirochete and latent rounded forms of both tested *Borrelia* sp. were cis‐2‐decenoic acid, baicalein, monolaurin and kelp (iodine). They demonstrated bacteriostatic and bactericidal effects in a time‐dependent manner against spiral and rounded forms. We are aware that fluorescence staining used in our study may not reveal few persisting rounded forms that may grow out from a population treated with the ‘drug of choice’; however, this would be relatively a small population. At the same time, these compounds, except kelp (iodine), revealed mild or moderate cytotoxic effect on human HepG2 cell line at their MBCs concentrations. However, no significant cytotoxic effects were observed at their MICs concentrations. In addition, while cis‐2‐decenoic acid, luteolin, baicalein, monolaurin and kelp (iodine) were effective against biofilm‐like colonies of *B. burgdorferi*, only baicalein and monolaurin showed significant reduction in biofilm of *B. garinii,* although at 1·5–2 times higher concentrations than those needed to induce bactericidal effect. This could be due to different biofilm morphologies, as *B. burgdorferi* forms more a colony‐like and scattered structure and *B. garinii* forms a layered and very condense assembly. These morphological differences might affect biofilm penetration by various compounds or signal different pathophysiological effects. Interestingly, doxycycline was effective against the spirochete form of both tested *Borrelia* sp. and displayed moderate effect against biofilm, but not against dormant rounded forms. These results correspond to findings reported by Sapi, *et al*. (Sapi *et al*. 2011).

Our results corroborate other findings on general antibacterial activity of the tested compounds and expand knowledge on their efficacy specifically against the *Borrelia* sp., for which there are only limited data. Several studies have shown that fatty acids and/or their derivatives, such as cis‐2‐decenoic acid and monolaurin, may play important antibacterial roles. Cis‐2‐decenoic acid is a short chain fatty acid belonging to a class of signaling molecules targeting cell membranes causing their disassembly and increased permeability. It was found to be bacteriostatic at 125–500 *μ*g ml^−1^ against methicillin‐resistant *Staphylococcus aureus* and its biofilm, without significant cytotoxicity on human dermal fibroblasts (Jennings *et al*. 2012). In separate studies, Davies and Marques observed dispersion of biofilms formed by *Pseudomonas aeruginosa* PAO1 by using cis‐2‐decenoic acid at a native concentration of 2·5 nmol l^−1^. A similar effect was observed on biofilms formed by other species, including *Escherichia coli*,* Klebsiella pneumoniae*,* Proteus mirabilis*,* Streptococcus pyogenes*,* Bacillus subtilis*,* Staph. aureus* and *Candida albicans* (Davies and Marques 2009). Another compound, monolaurin, has been shown to inhibit growth and induce biofilm dispersion of a broad spectrum of Gram‐positive and Gram‐negative bacteria at concentrations of 30–500 *μ*g ml^−1^ (Schlievert *et al*. 1992) without cytotoxic effects on human umbilical vein endothelial cells (HUVECs) when used up to 100 *μ*g ml^−1^ over a 2‐week period. Also, a study carried out by Batovska, *et al.,* revealed antibacterial activity of monolaurin against several Gram‐positive strains such as *Staphylococcus* sp., *Corynebacterium* sp., *Bacillus* sp., *Listeria* sp. and *Streptococcus* sp. (Batovska *et al*. 2009). Monolaurin is the ester of glycerol and dodecanoic acid, a medium chain fatty acid. As a lipophilic compound it may disrupt cell membranes, and thus similar to cis‐2‐decenoic acid, it may act as a destabilizer of the biofilm matrix (Kabara *et al*. 1973).

Baicalein, another compound tested in our study, belongs to flavonoids with phenolic groups in their structure. Yun, *et al.,* have indicated that baicalein's antibacterial activity against *Staph. aureus* may include increasing penetrability of bacterial membranes, inhibiting protein synthesis and affecting activities of succinate dehydrogenase (SDH), malate dehydrogenase (MDH) and DNA topoisomerase I and II (Yun *et al*. 2012). Others have demonstrated that baicalein could reverse the ciprofloxacin resistance of methicillin‐resistant *Staph. aureus* by possibly causing ATP deficiency (Chan *et al*. 2011). It can act either alone (at a concentration of 256 *μ*g ml^−1^) or synergistically (at a concentration of 32 *μ*g ml^−1^) with gentamicin against vancomycin‐resistant *Enterococcus* isolates (Chang *et al*. 2007). In addition, Moghaddam *et al.,* noticed that baicalein had no cytotoxic effect on Vero cells up to 62 *μ*g ml^−1^, and at 125–250 *μ*g ml^−1^, it decreased the survival of these cells by 20–25% (Moghaddam *et al*. 2014). In additionWang *et al*. (2014), observed no effect of baicalein below 100 *μ*mol l^−1^ on the viability of HeLa cells.

One of the best antibacterial, antifungal and antiviral agents still considered is iodine, due to its rapid penetration of phospholipid membranes and its oxidative damage to bacterial proteins, nucleotides and fatty acids (McDonnell and Russell 1999). Iodine is also reported as a relatively safe compound (York *et al*. 1988; Slots 2002). The study of Wichelhaus, *et al.,* has shown the antibacterial effects of povidone‐iodine at concentrations between 1–10% against highly resistant Gram‐positive bacteria (Wichelhaus *et al*. 1998). Soares, *et al.,* established the MICs for povidone‐iodine against dermatophytes isolated from patients with tinea pedis that ranged from 4 to 128 *μ*g ml^−1^ (Soares and Cury 2001). In addition, Kaspar, *et al*. reported that a povidone‐iodine concentration below 0·2% had no significant effect on proteoglycan and DNA synthesis of chondrocytes as well as viability and proliferation of BALB3T3 fibroblasts (Kaspar *et al*. 2006).

In summary, this study fills the gap in scientific data regarding the efficacy of select phytochemicals against active and latent forms of *B. burgdorferi* and *B. garinii*. This comprehensive evaluation of 15 natural compounds shows that baicalein and monolaurin were effective against all morphological forms of both tested species of *Borrelia*, which might indicate the importance of phenyl groups and fatty acids in the anti‐borelieae activity. Their effective concentrations were within the same ranges as reported earlier for other bacterial species and showed no or moderate cytotoxicity against human HepG2 cells.

## Conflict of Interest

No conflict of interest declared.
